# Leukemia cutis: a rare dermatological presentation of leukemia

**DOI:** 10.11604/pamj.2024.48.69.43631

**Published:** 2024-06-27

**Authors:** Hemant Juneja, Gaurang Aurangabadkar

**Affiliations:** 1 Department of General Medicine, Datta Meghe Medical College, Nagpur, Datta Meghe Institute of Higher Education and Research, Deemed University, Sawangi (Meghe), Wardha,; 2 Department of Respiratory Medicine, Datta Meghe Medical College, Nagpur, Datta Meghe Institute of Higher Education and Research, Deemed University, Sawangi (Meghe), Wardha

**Keywords:** Leukemia cutis, acute myeloid leukemia, skin biopsy

## Image in medicine

Leukemia cutis is defined as the infiltration of malignant leucocytes or the cell precursors into the dermis, epidermis, or subcutis, leading to prominently identifiable dermatological lesions. Leukemia cutis may be seen in conjunction, preceding or following a clinical diagnosis of systemic leukemia. The presence of leukemia cutis is often a marker of poor prognosis and often denotes the presence of extramedullary metastases. We present the clinical image of a 45-year-old patient who presented with chief complaints of continuous high-grade fever with easy fatiguability, dyspnea on exertion along with prominent multiple nodular lesions on the face with symmetrical involvement for 15 days. For further evaluation to assess the possible etiology of the illness, a dermatologist's opinion was sought and a skin biopsy revealed diffuse dense infiltrates of neoplastic cells within the reticular dermis. The reporting histopathologist gave a diagnosis of leukemia cutis. A bone marrow aspiration of the patient was done and a sample was sent for flow cytometry analysis, which was suggestive of Acute myeloid leukemia with monocytic differentiation. After the diagnosis of Acute myeloid leukemia, a specialist opinion was taken from the Oncologist, and the patient was started on a chemotherapy regimen and asked to review for follow-up after 1 month.

**Figure 1:**
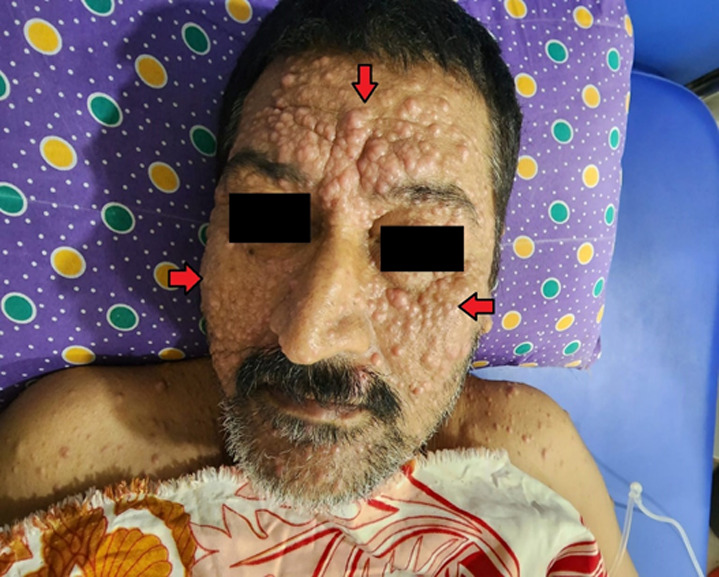
presence of bilateral nodular lesions on the face with a symmetrical distribution

